# 30-Day Postoperative Outcomes in Adults with Obstructive Sleep Apnea Undergoing Upper Airway Surgery

**DOI:** 10.3390/jcm11247371

**Published:** 2022-12-12

**Authors:** Samuel Knoedler, Leonard Knoedler, Helena Baecher, Martin Kauke-Navarro, Doha Obed, Cosima C. Hoch, Yannick F. Diehm, Peter S. Vosler, Ulrich Harréus, Ulrich Kneser, Adriana C. Panayi

**Affiliations:** 1 Department of Plastic, Hand and Reconstructive Surgery, University Hospital Regensburg, 93053 Regensburg, Germany; 2 Division of Plastic Surgery, Department of Surgery, Brigham and Women’s Hospital, Harvard Medical School, Boston, MA 02115, USA; 3 Division of Plastic and Reconstructive Surgery, Massachusetts General Hospital, Harvard Medical School, Boston, MA 02114, USA; 4 Department of Surgery, Division of Plastic Surgery, Yale New Haven Hospital, Yale School of Medicine, New Haven, CT 06510, USA; 5 Department of Plastic, Aesthetic, Hand and Reconstructive Surgery, Hannover Medical School, 30625 Hannover, Germany; 6 Department of Otolaryngology, Head and Neck Surgery, Rechts der Isar Hospital, Technical University Munich, 81675 Munich, Germany; 7 Department of Hand-, Plastic and Reconstructive Surgery, Microsurgery, Burn Trauma Center, BG Trauma Center Ludwigshafen, University of Heidelberg, 67071 Ludwigshafen, Germany; 8 Head and Neck Cancer Center, Sarasota Memorial Health Care System, Sarasota, FL 34239, USA; 9 Department of Otolaryngology, Head and Neck Surgery, Asklepios Hospital, 83646 Bad Toelz, Germany

**Keywords:** obstructive sleep apnea (OSA), airway surgery, head and neck surgery, big data

## Abstract

Background: Obstructive sleep apnea (OSA) is a chronic disorder of the upper airway. OSA surgery has oftentimes been researched based on the outcomes of single-institutional facilities. We retrospectively analyzed a multi-institutional national database to investigate the outcomes of OSA surgery and identify risk factors for complications. Methods: We reviewed the American College of Surgeons National Surgical Quality Improvement Program (NSQIP) database (2008–2020) to identify patients who underwent OSA surgery. The postoperative outcomes of interest included 30-day surgical and medical complications, reoperation, readmission, and mortality. Additionally, we assessed risk-associated factors for complications, including comorbidities and preoperative blood values. Results: The study population included 4662 patients. Obesity (n = 2909; 63%) and hypertension (n = 1435; 31%) were the most frequent comorbidities. While two (0.04%) deaths were reported within the 30-day postoperative period, the total complication rate was 6.3% (n = 292). Increased BMI (*p* = 0.01), male sex (*p* = 0.03), history of diabetes (*p* = 0.002), hypertension requiring treatment (*p* = 0.03), inpatient setting (*p* < 0.0001), and American Society of Anesthesiology (ASA) physical status classification scores ≥ 4 (*p* < 0.0001) were identified as risk-associated factors for any postoperative complications. Increased alkaline phosphatase (ALP) was identified as a risk-associated factor for the occurrence of any complications (*p* = 0.02) and medical complications (*p* = 0.001). Conclusions: OSA surgery outcomes were analyzed at the national level, with complications shown to depend on AP levels, male gender, extreme BMI, and diabetes mellitus. While OSA surgery has demonstrated an overall positive safety profile, the implementation of these novel risk-associated variables into the perioperative workflow may further enhance patient care.

## 1. Introduction

Obstructive sleep apnea (OSA) is a chronic disorder defined as increased pharyngeal airway resistance during sleep with subsequent repetitive collapse of the upper airway [1,2]. With over one billion people affected worldwide, OSA represents a highly prevalent and continually increasing disorder. OSA patients suffer from different symptoms, including sleep fragmentation, hypoxia, and increased cardiovascular morbidity [3,4,5,6].

Positive airway pressure (PAP) therapy represents the gold standard in non-surgical OSA management [7]. A wide array of surgical treatment options exist, including (i) uvulopalatopharyngoplasty (UPPP); (ii) other soft tissue reduction procedures, such as tonsillectomy, glossectomy, and epiglottidectomy; (iii) skeletal surgeries, such as maxillomandibular advancement (MMA), genioglossus advancement (GA), and hyoid myotomy and suspension (HMS); and (iv) upper airway bypass procedures, including tracheostomy for severe OSA [1,3].

In most cases, OSA surgeries are performed in non-academic facilities [8]. Outcome research on complication rates and risk-associated factors for OSA is often derived from retrospective analyses of single-surgeon, single-institution, or technique-specific medical records, which can reduce research transferability and significance to the scientific community [9]. By pooling patient data with geographical and institutional variation, an analysis of multicenter national databases can help identify more robust risk-associated factors and provide a panoramic view of postoperative outcomes in OSA patients.

The American College of Surgeons (ACS) National Surgical Quality Improvement Program (NSQIP) provides an extensive and diverse patient cohort by collecting validated data from more than 700 US hospitals. We, therefore, query this database to fill the research gap regarding the outcomes and occurrence of adverse events of OSA procedures in larger, mostly academic hospital centers, which may represent more complex cases with a multimorbid patient group [10].

## 2. Methods

### 2.1. Data Source and Patient Selection

Data were collected between 2008 and 2020 from the American College of Surgeons National Surgical Quality Improvement Program (ACS-NSQIP) database. As a multi-institutional catalog, the ACS-NSQIP records over 150 pre-, peri-, and postoperative data points. Since the records analyzed did not contain patient-identifying information, the study was exempt from Institutional Review Board approval.

The ACS-NSQIP database was queried to identify all patients who underwent surgical treatment for obstructive sleep apnea (OSA). Specifically, 13 annual records between 2008 and 2020 were searched for ICD-9-CM 327.23 (“Obstructive sleep apnea”) and ICD-10-CM G47.33 (“Obstructive sleep apnea”) codes. In a second step, we screened this OSA cohort of 4781 cases and retrieved all cases in which bariatric surgical procedures were performed. We excluded a total of 119 cases of bariatric treatment to obtain a more homogeneous cohort undergoing head and neck surgery as the only therapeutic management for OSA. Thus, the analyzed cohort did not include any case of bariatric OSA treatment, either as the main procedure or as a concomitant procedure. Finally, the generated patient pool was manually cross-checked by two investigators (S.K. and A.C.P.), and the classification as head and neck OSA surgery was confirmed for each individual case. A third investigator (L.K.) was consulted in cases of discrepant assessments, with any unclear records being excluded from the analysis.

### 2.2. Variable Extraction

Pre-, peri-, and thirty-day postoperative variables were extracted for analysis.

(i) Preoperative data were evaluated as follows: (a) patient demographics (sex, age, race, height in inches, and weight in pounds), (b) comorbidities (history of chronic obstructive pulmonary disease (COPD) or congestive heart failure (CHF), active dialysis treatment, diabetes mellitus, hypertension, dyspnea, metastatic cancer, smoking status in the past year, steroid or immunosuppressive therapy use, weight loss greater than 10% of body weight, wound infections, ventilator dependency, and functional health status), (c) preoperative scores (wound classification (score of 1–4) and American Society of Anesthesiology (ASA) physical status classification (score of 1–5)), and (d) preoperative laboratory values, including serum sodium, blood urea nitrogen (BUN), serum creatinine, serum albumin, total bilirubin, serum glutamic-oxaloacetic transaminase (SGOT), alkaline phosphatase (ALP), white blood count (WBC), hematocrit, platelet count, partial thromboplastin time (PTT), international normalized ratio (INR), and prothrombin time (PT). In addition, we calculated the body mass index (BMI) for all patients using the following formula: weight (pounds)/height (inches)2 × 703. All extracted preoperative variables are shown in Table 1.

(ii) In terms of perioperative data, we evaluated the type of anesthesia (general, monitored, epidural or spinal, local or regional, and other), surgical specialty (otolaryngology, general surgery, and other), setting (inpatient or outpatient), year of surgery within the 13-year period of 2008–2020, and total operative time in minutes. All perioperative data are shown in Table 2 and Table 3.

For an in-depth evaluation, we manually analyzed all cases of head and neck OSA surgery and first classified them into one of the following types of surgery (based on the most invasive procedure or the entered main procedure): uvulopalatopharyngoplasty (UPPP), palatopharyngoplasty (PPP), tonsillectomy, uvulectomy, partial glossectomy, genioglossus advancement, maxillomandibular advancement, hyoid myotomy and suspension, cases including craniofacial osteotomy, cases including epiglottidectomy, cases including tracheostomy, and other. Next, we refined this classification system by specifying which concomitant (less invasive) procedures were entered in parallel.

**Figure 1:**
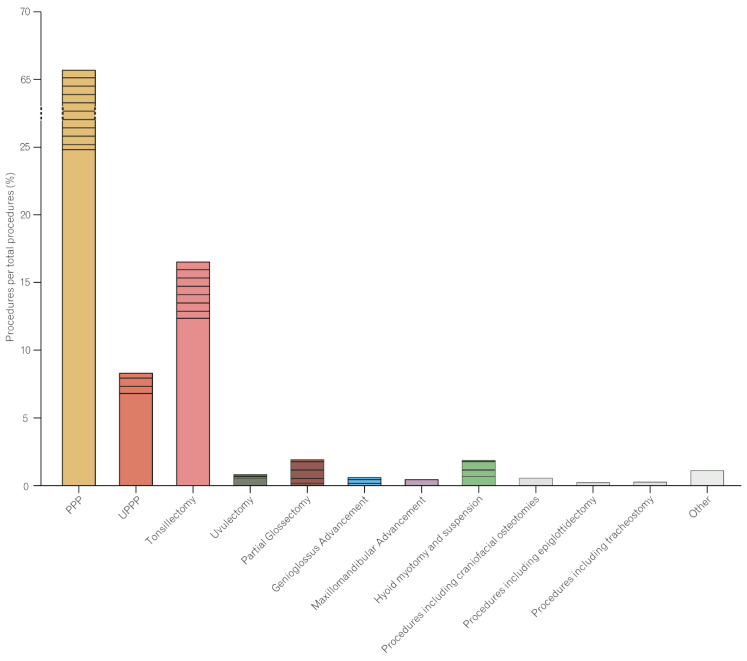
Procedure distribution. Procedures performed concomitantly with other procedures (combined procedures) are shown with a striped pattern. The majority of procedures (>65%) were PPPs, followed by tonsillectomies. The exact numbers are shown in Table 3. PPP, palatopharyngoplasty; UPPP, uvulopalatopharyngoplasty.

When classifying and labeling the individual types of surgery, we closely adhered to the nomenclature recorded in the NSQIP database. Accordingly, the specification of the surgical types was based on the procedural description and the recorded Current Procedural Terminology (CPT) codes. Further, we followed the guidelines of the American Society of Anesthesiologists Task Force on the management of patients with OSA in assessing the invasiveness of the procedures [11]. In rare cases, for example, craniofacial osteotomies, a more precise specification was not possible due to limited case information. Surgical characteristics, including the classification pattern and the prevalence of each (sub)type of surgery, are summarized in Table 3 and Figure 1.

(iii) The gathered and analyzed 30-day postoperative outcomes included discharge destination (home, not home, and other or unknown) and length of hospital stay (LOS). LOS was counted as the difference in days between the date of admission and the date of discharge. Any complication was defined as the occurrence of any of the following: mortality, reoperation, readmission or unplanned readmission, and surgical or medical complications. For further analyses, all surgical complications reported in the ACS-NSQIP database (i.e., superficial and deep incision-site infections, organ-space infections, wound lacerations or dehiscences, and blood transfusions) that arose at least once were considered. Similarly, while evaluating all medical complications captured in the ACS-NSQIP catalog (i.e., pulmonary embolism, pneumonia, reintubation, ventilator use for more than 48 h, infection of the urinary tract, renal insufficiency, acute renal failure, deep-vein thrombosis or thrombophlebitis, cardiac arrest, cerebrovascular incident or stroke, myocardial infarction, sepsis, and septic shock), we focused on those for which at least one case was reported. Detailed information on postoperative outcomes after head and neck OSA surgery is listed in Table 4, Table 5 and Table 6.

Of note, as the primary composite outcome, we defined the occurrence of any complication, i.e., mortality, reoperation, readmission, unplanned readmission, any surgical complication, or any medical complication. In this context, it is important to mention that we counted the total number of patient cases and not the sheer number of complications. In other words, if a patient both returned to the operating room and experienced a medical complication, this was recorded as any complication n = 1. Second, we analyzed all individual outcomes separately and determined the mean length of hospital stay. To this end, we evaluated the frequency of mortality, reoperation, (unplanned) readmission, any surgical, and any medical complication. The latter two included the occurrence of superficial or deep incisional infection, organ-space infection, dehiscence, pneumonia, reintubation, pulmonary embolism, ventilator use for more than 48 h, myocardial infarction, cardiac arrest, deep-vein thrombosis or thrombophlebitis, urinary tract infection, sepsis, and septic shock.

### 2.3. Statistical Analysis

Data were collected and stored in an electronic laboratory notebook (LabArchives, LLC, San Marcos, CA, USA) and evaluated with GraphPad Prism (V9.00 for MacOS, GraphPad Software, La Jolla, CA, USA). Continuous variables (i.e., age and BMI) were analyzed with independent *t*-tests and reported as means with standard deviations. Pearson’s chi-squared test was used to measure differences in categorical variables. In cases with fewer than 10 events, Fisher’s exact test was applied. Statistical significance was measured at *p* < 0.05. a univariable subgroup analysis was performed to accomplish risk-associated factors for complications by separating the cohort into three groups depending on the occurrence of any, surgical, or medical complications. An in-depth statistical analysis was conducted using ordinary least squares (OLS) based on a multivariate logistic regression analysis. OLS regression is a statistical-mathematical method calculating the association between one or more independent variables and a dependent variable. Multivariate regression is considered an advanced version of normal OLS regression. These models were performed to control for confounding by including all variables found to be significant risk-associated factors for the occurrence of any, surgical, or medical complications. More specifically, this analysis was adjusted for gender, BMI, setting, diabetes, hypertension, and ASA physical status classification (any complications); for age, race, setting, COPD, and dyspnea (surgical complications); and for BMI, setting, diabetes, obesity, corticosteroid use, ASA physical status classification, and functional status (medical complications).

## 3. Results

### 3.1. Patient Demographics

The study population included 4662 patients who underwent OSA surgery over a 13-year review period (2008–2020). The average patient age was 42 ± 13, while male (n = 3388; 73%) and white (n = 2979; 64) patients with class-1 or -2 obesity (BMI: 33 ± 7.7) accounted for the majority of OSA surgery candidates. Obesity (n = 2909; 63%) and hypertension (n = 1435; 31%) were the most frequent comorbidities. In our study population, 16% (n = 742) declared to be current smokers. Detailed demographic data and comorbidities of the entire study population are described in Table 1. Supplementary Table S1 focuses on patients who underwent isolated uvulopalatopharyngoplasty, palatopharyngoplasty, and tonsillectomy procedures and provides a breakdown of their characteristics.

### 3.2. Surgical Characteristics

Isolated palatopharyngoplasty (PPP) (n = 1161; 25%) was the most frequently performed surgery, with tonsillectomy (PPP with tonsillectomy) (n = 887; 19%) and turbinate reduction (PPP with turbinate reduction) (n = 409; 8.8%) as most common multilevel procedures. Most procedures were performed in an outpatient setting (n = 3280; 70%). Table 2 and Table 3 display surgical characteristics in detail.

### 3.3. Perioperative Outcomes

The mean operation time was 66 ± 54 min. After a postoperative LOS of 0.9 ± 2.0 days on average, 84% (n = 3956) of patients were discharged home (Table 4).

### 3.4. Postoperative Surgical and Medical Outcomes

The occurrence of any complication (i.e., mortality, reoperation, readmission, unplanned readmission, any surgical complication, or any medical complication) was recorded in 292 patient cases (6.3%) (Table 4). While two (0.04%) deaths were reported within the 30-day postoperative period, the reoperation rate amounted to 3.5% (n = 163). The surgical complication rate was 1.0% (n = 48), with superficial incisional infection (n = 26; 0.6%) as the most frequently reported adverse surgical event. Medical complications occurred in 55 (1.2%) cases, of which pneumonia constituted 21 (0.5%) cases. Male sex (*p* = 0.03), increased BMI (*p* = 0.01), inpatient setting (*p* < 0.0001), history of diabetes (*p* = 0.002), hypertension requiring treatment (*p* = 0.03), and ASA scores ≥ 4 (*p* < 0.0001) were identified as risk-associated factors for the occurrence of any postoperative complications. Advanced age (*p* = 0.04), inpatient setting (*p* = 0.0002), history of COPD (*p* = 0.003), and dyspnea (*p* = 0.03) were identified as risk-associated factors for the occurrence of any surgical complication. In terms of medical complications, increased BMI (*p* < 0.0001), inpatient setting (*p* < 0.0001), history of diabetes (*p* < 0.0001), corticosteroid use (*p* = 0.01), and ASA scores ≥ 4 (*p* = 0.0006) were identified as risk-associated factors. A multivariable analysis confirmed that ASA score and diabetes were independent risk-associated factors for the occurrence of any complication (*p* = 0.03 and *p* = 0.001, respectively; Table 7). Further details about the multivariable assessments of any, surgical, and medical complications are described in Table 7 and Table 8. Increased alkaline phosphatase (ALP) was identified as a risk-associated factor for the occurrence of any complication (*p* = 0.02) and medical complications (*p* = 0.001). Detailed preoperative lab value data are described in Table 9.

**Figure 2:**
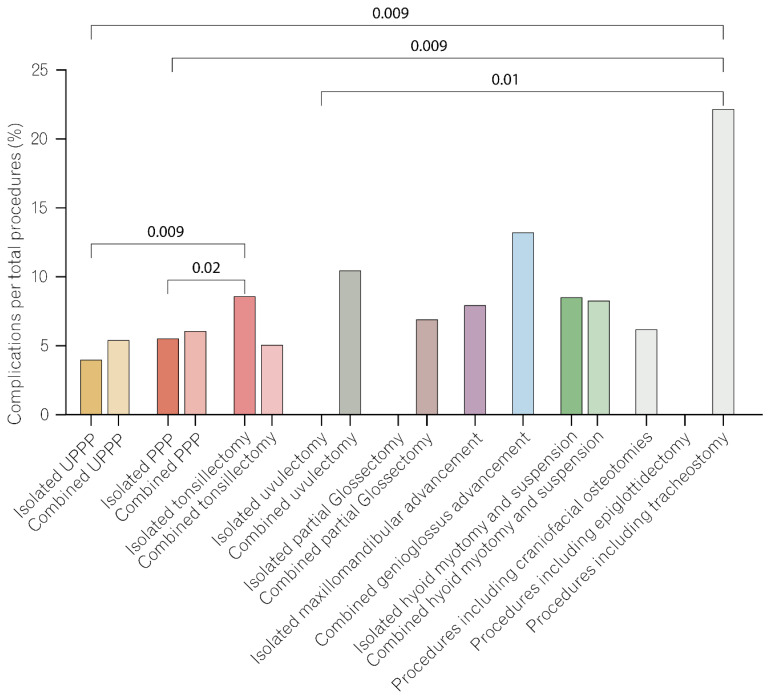
Complications rates for different procedures. Isolated and combined procedures did not significantly differ in terms of complication rates. Tonsillectomy, when performed as an isolated procedure, had a significantly higher rate of complications than isolated UPPP or isolated PPP. Tracheostomy procedures had the highest rate of complications, significantly higher than isolated UPPP, isolated PPP, and isolated uvulectomy. The exact numbers are shown in Supplementary Tables S2 and S3.

To delineate a correlation pattern between the type of procedure performed and the occurrence of adverse events, we first reviewed the total number of complications. We found that most complications occurred in patients who underwent palatopharynoplasty (PPP), (n = 65) followed by patients receiving tonsillectomy (n = 50) (Table 5). This was not surprising considering that these two surgical procedures also numerically accounted for the largest proportion of the cohort (Figure 1). We, therefore, calculated a complication rate (defined as the number of complications within a surgery type relative to the total number of patients who underwent that specific procedure; Table 5). A comparison of complication rates among the different (sub)types of surgery revealed that there were no statistically significant differences between isolated procedures and multilevel surgeries (Supplementary Table S2). When focusing exclusively on isolated procedures, we found isolated tonsillectomy to be associated with a significantly higher risk than isolated uvulopalatopharyngoplasty (*p* = 0.009) and isolated palatopharyngoplasty (*p* = 0.02; Supplementary Table S3). Further, tracheostomy showed significantly higher complication rates than isolated uvulopalatopharyngoplasty (*p* = 0.009), isolated palatopharyngoplasty (*p* = 0.02), and isolated uvulectomy (*p* = 0.01; Figure 2 and Supplementary Table S3). Comparable trends were also noticeable in the multivariable assessment of complication occurrences among the different (sub)types of surgery. While isolated tonsillectomy was found to be statistically significantly associated with any complications (*p* = 0.003), tracheostomy surgery was associated with significantly higher risks for any (*p* = 0.04) and medical complications (*p* = 0.04; Table 8). The multivariable analysis suggested two surgical combinations as high-risk conditions for the occurrence of postoperative complications. Namely, combined genioglossus advancement and maxillomandibular advancement, as well as combined palatopharyngoplasty and tonsillectomy, were frequent among the risk-associated types of surgery (Table 8).

## 4. Discussion

Big databases represent a powerful tool for tracking surgical outcomes and improving patient care. For OSA patients, as a vulnerable patient group per se, it is important for surgeons to thoroughly determine a patient’s risk profile prior to undergoing surgery [12]. We queried the ACS-NSQIP database to investigate medical and surgical complications and risk-associated factors, as well as 30-day postoperative outcomes, in 4662 OSA surgery cases.

For the purpose of a homogeneous patient cohort (and, thus, for better interindividual comparability), we excluded all cases of bariatric surgery as a therapeutic approach for OSA. Nonetheless, in our study population undergoing head and neck surgery, middle-aged (42 ± 13 years of age) males with class-1 obesity (BMI: 33 ± 7.3 kg/m^2^) still represented the most common OSA patient. This finding aligns with recent studies by Du et al. and Zaghi et al. who each investigated demographic patterns in OSA surgery [13,14]. Further, we found that 62% of patients undergoing upper airway surgery suffered from obesity and 31% from hypertension, while 16% of patients were current smokers. The high prevalence of comorbidities, such as obesity, was also reported in a 2014 Korean population study of 348 patients, as well as in a 2015 study by Heinzer et al. including 2121 OSA patients [15,16]. The high prevalence of nicotine abuse in OSA patients has also been described in the scientific literature [17,18]. Our findings, therefore, underscore the vulnerability of this specific patient group (i.e., middle-aged, male, and obese patients with a medical record of nicotine abuse) to OSA. With the background of increasing worldwide obesity rates and persistently high smoking prevalence, joint efforts are needed to sensitize and target risk patients, such as overweight adolescents or current smokers, before the clinical manifestation of OSA symptoms [19,20,21].

Surgical OSA therapy is a case-to-case decision based upon individual anatomical findings with the possible combination of different surgical techniques in multilevel surgeries [2,22]. Of note, UPPP, which was considered the standard OSA surgery prior, has been successively replaced by multilevel surgery to simultaneously address variables that predispose patients to UPPP-resistant OSA (e.g., narrowing or collapse at sites other than the retropalate) [23]. The multilevel approach renders perioperative risk evaluation more challenging and necessitates procedure-adjusted complication assessment. In our study, isolated UPPP accounted for 6.9% of the cases, while palatopharyngoplasty combined with different concurrent procedures accounted for 40% of the patients (Figure 1). This distribution pattern corroborates the ongoing shift toward concomitant surgeries in OSA patients.

Regarding complication rates, different studies have shown overall perioperative complication rates in OSA surgery ranging between 1.0% and 15% [22,24]. More recent reports have indicated relatively low complication rates for OSA surgery: while van Daele et al. documented reoperation rates and surgical site infections in 4.8% and 0.9% of all cases, respectively, Rosero et al. found postoperative complications occurring in 6.4% of surgical OSA patients [25,26]. Both authors reported surgery-related mortality rates of less than 0.1%. Overall, these numbers are comparable to our analysis, where we found 2 cases of death (0.4%), 292 (6.3%) cases of any complications, and 163 reoperations (3.5%) due to complications.

Interestingly, in our study, no significant differences in the occurrences of complications and the incidences of readmissions and reoperations were found between multilevel surgeries and isolated procedures (Supplementary Table S2). The stacking of concomitant procedures for customized patient therapy was not associated with a significantly increased risk. This finding is in line with recent studies that have highlighted the safety profile of multilevel OSA surgery [27,28,29,30,31]. More specifically, the SAMS randomized clinical trial involved 51 patients undergoing multilevel OSA surgery (i.e., uvulopalatopharyngoplasty and minimally invasive tongue volume reduction), with only two participants (4%) experiencing postoperative adverse events [30]. Similarly, Bosco et al. reported the absence of any unexpected complications during the postoperative follow-up of 24 patients receiving one-stage multilevel OSA surgery (pharyngoplasty, tongue base reduction, or partial epiglottectomy) [31]. In 2021, a meta-analysis evaluated 37 studies and a total of 1639 patients undergoing multilevel OSA surgery. With major complications being reported in only 1.1% of all cases, this analysis further validated the procedures’ safety [29]. Thus, our study substantiates the current trend towards multilevel OSA surgery also from a risk-associated standpoint. However, we identified two surgical combinations (i) genioglossus advancement plus concomitant maxillomandibular advancement and ii) palatopharyngoplasty plus parallel tonsillectomy) as high-risk procedures. Therefore, although this study may encourage surgeons to consider concomitant procedures for more tailored surgical management of OSA patients, surgeons should pay particular attention to these two combinations during preoperative planning and critically evaluate patients’ eligibility. In this context, we advocate an individualized case-by-case decision that takes into account both the patient characteristics and the relevant circumstances (such as psychosocial support, monetary burden, and available (mid- and long-term) nursing). The proposed predictive factors, including patient gender, body weight, comorbidities, and laboratory values, may aid in achieving an evidence-based risk assessment.

Of note, tracheostomy was also identified as a risk-associated factor for both any and medical complications, regardless of the main surgical procedure. In general, tracheostomy has been shown to be a safe airway management technique in most cases, as well as a potential, yet uncommon, therapeutic alternative for patients with severe OSA [32,33,34,35,36]. Still, tracheostomy is an invasive procedure that carries a risk of high morbidity. The spectrum of potential adverse events is broad, ranging from postoperative bleeding and infection to tracheal wall injury and tube obstruction. Further tracheostomy-related complications (such as stenosis, malacia, and fistula formation) may also be life-threatening by affecting airway passability, ultimately rendering this procedure one of the most morbid in the wide field of OSA therapy [37,38,39]. Koitschev et al. showed that the surgical technique used for tracheotomy influenced the risk for tracheostomy-related complications. They found that surgical approaches resulting in an epithelialized tracheostoma minimized the risk for tracheostomy-related complications [40]. To our knowledge, this is the first study to outline the increased risk of tracheostomy in OSA surgery. Therefore, surgeons may critically weigh the potential benefits of tracheostomy, such as enhanced nursing care, against the additional complication risk in OSA patients. However, this finding should be corroborated on larger scales.

OSA patients represent a vulnerable patient group in surgical risk management [12,41]. Therefore, the identification of risk-associated factors plays a pivotal role in enhancing patient care. In our analysis, we found male gender, diabetes mellitus, and ASA scores ≥ 4 to be predictive of any complications. While these factors have been described as risk-associated factors in other fields of surgery, this is the first study to reveal an increased risk for this patient profile when undergoing OSA surgery [42,43,44,45,46,47,48]. For medical complications, we not only found extreme obesity (i.e., BMI > 40 kg/m^2^) but also underweight status (i.e., BMI < 18.5 kg/m^2^) to be significant risk-associated factors (Table 7; Supplementary Figure S1). While Du et al. found the same for OSA surgery patients in obesity classes 2 and 3 when analyzing 1923 OSA surgery cases, this is the first study to reveal an increased risk of medical complications in underweight patients who underwent OSA surgery. Further, we identified elevated alkaline phosphatase (ALP) values to be predictive of medical and surgical complications following OSA surgery. Increased ALP levels can be due to hepatic and biliary diseases, as well as bone disorders [49,50]. Increased ALP values have been implicated with elevated complication rates in other types of surgery, but the exact pathomechanism by which ALP influences perioperative patient health remains to be determined [51,52]. Based on our analysis, we propose that particular attention be paid to patients with ALP levels lower than 81.5 U/L given the association with postoperative complications.

Although the experience, dexterity, and expertise of the surgeon can have a substantial impact on therapeutic success, a series of pre- and perioperative measures can help optimize patient safety. Care providers may, therefore, wish to implement the identified risk-associated factors into their clinical workflow to optimize (i) patient counseling, (ii) high-risk patient group identification, (iii) preoperative patient-tailored planning, (iv) perioperative multimodal monitoring, and (v) postoperative (long-term) follow-up. Thus, by updating and upgrading the risk assessment armamentarium, an individual surgeon can contribute to further honing surgical OSA management.

### Limitations

To the best of our knowledge, the study is the first to analyze risks, complications, and outcomes after different kinds of surgical treatment in OSA patients based on multi-center data collected over more than a decade. However, interpretation of the findings considering the study’s limitations is mandatory. First, we would like to emphasize that we analyzed correlations and not causalities with the statistical calculations. We identified factors that were associated with higher perioperative risk. However, the underlying causal mechanisms remain to be elucidated. In general, the NSQIP database only provides a limited postoperative follow-up for a 30-day period, meaning that long-term complications, e.g., disease recurrence, remain uncovered [53]. Further, this study entails the risk of unconsidered bias and confounding factors due to its conceptualization as a retrospective data analysis. Due to subjectivity and the unequal expertise of professional database documentation, intra- and interinstitutional differences in the precision and completeness of data collection represent additional limitations in data comparison [54]. However, the robustness, quality, and validity of the entered information are warranted by spot audits and peer controls [55,56]. In fact, according to Shiloach et al., the NSQIP database established low variance in heterogeneity by means of trained data collectors and ongoing audits of data reliability [57].

The range of OSA therapy is far-reaching. The ACS-NSQIP does not cover all of the available treatment modalities [25]. As such, e.g., the implantation and use of a hypoglossal nerve stimulator device (“upper airway stimulation system”) is not included in this study. Protocols and therapeutic approaches in the surgical management of OSA vary across the globe [58,59,60,61]. Since the NSQIP is a national US database, the transferability of our findings is limited to the American healthcare system [62]. Lacking information about significant subgroup variables, including OSA severity and primary OSA treatment, may lead to deviating outcomes in patient cases. Not considering the initial severity of OSA and the corresponding degree of invasiveness of the surgical procedure, complication rates constitute a kind of average value without the possibility of exact procedure identification. The NSQIP database does not provide information regarding the improvement of OSA symptoms after surgery; thus, no conclusions regarding the impact of risk-associated factors on surgical success rates could be drawn. Furthermore, the ACS-NSQIP database does not specify the criteria upon which an OSA diagnosis is generated and validated. Thus, it cannot be ascertained whether overnight polysomnography was used as a diagnostic procedure. Nevertheless, despite the aforementioned limitations, we are convinced of the study’s significance, validity, and value. The described findings may help further refine the perioperative protocols of OSA surgery and, ultimately, optimize patient care.

## 5. Conclusions

Utilizing the NSQIP-ACS big database, we analyzed 4662 patients undergoing OSA surgery over a 13-year period. We identified that elevated ALP levels (≥81.5 U/L), male gender, diabetes mellitus, values at extreme ends of the BMI scale (BMI < 18.5 kg/m^2^ and >40 kg/m^2^), and ASA scores ≥ 4 were predictive factors for postoperative complications. Awareness of these risk-associated factors may help surgeons carefully balance a patient’s eligibility and refine their perioperative management. More specifically, by accounting for these factors during the preoperative planning stage, high-risk patients can be preemptively identified and closely monitored. Moreover, we noted no significant differences in the safety profiles between multilevel surgeries and isolated interventions. While these findings generally imply a step toward treatment individualization, we revealed two (relatively) high-risk surgical combinations (i.e., genioglossus advancement plus concomitant maxillomandibular advancement and palatopharyngoplasty plus parallel tonsillectomy). In addition, cases involving tracheostomy were found to be associated with an increased incidence of adverse events. OSA surgeons should be mindful of these correlations when planning individual treatments and counseling patients.

## Figures and Tables

**Table 1:** Patient demographics and comorbidities. Reported as n (%), unless otherwise stated.

Characteristic	Patients (n = 4662)
**Demographics**	
Sex	
Female (n)	1273 (27)
Male (n)	3388 (73)
Age, mean ± SD	42 ± 13
BMI, mean ± SD	33 ± 7.3
**Race**	
American Indian or Alaskan Native	28 (0.6)
Asian	244 (5.2)
Native Hawaiian or Pacific Islander	51 (1.1)
Black or African American	544 (12)
White	2979 (64)
Other or unknown	804 (17)
**Preoperative health and comorbidities**	
Diabetes	469 (10)
Insulin-treated diabetes	137 (2.9)
COPD	60 (1.3)
CHF	5 (0.1)
Obesity	2909 (62)
Hypertension	1435 (31)
Dyspnea	229 (4.9)
Current smoker	742 (16)
Corticosteroid use	80 (1.7)
Wound infection	13 (0.3)
**ASA physical status classification score**	
1—No disturbance	217 (4.7)
2—Mild disturbance	2650 (57)
3—Severe disturbance	1744 (37)
4—Life-threatening	45 (1.0)
**Wound class**	
1—Clean	179 (3.8)
2—Clean/contaminated	4396 (94)
3—Contaminated	63 (1.4)
4—Dirty/infected	24 (0.5)
**Functional Status**	
Independent	4606 (99)
Partially or totally dependent	56 (1.2)

ASA, American Society of Anesthesiology.

**Table 2:** Surgical characteristics. Reported as n (%), unless otherwise stated.

Characteristic	Patients (n = 4662)
**Surgical specialty**	
General	39 (0.8)
ENT	4587 (98)
Other	36 (0.8)
**Type of anesthesia**	
General	4642 (100)
Local	3 (0.06)
Monitored anesthesia care	8 (0.2)
Epidural or spinal	6 (0.1)
Other or unknown	3 (0.06)
**Setting**	
Inpatient	1382 (30)
Outpatient	3280 (70)
**Year of surgery**	
2008	173 (3.7)
2009	202 (4.3)
2010	306 (6.6)
2011	120 (2.6)
2012	441 (9.5)
2013	429 (9.2)
2014	440 (9.4)
2015	455 (9.8)
2016	457 (9.8)
2017	516 (11)
2018	445 (9.5)
2019	376 (8.1)
2020	302 (6.5)

**Table 3:** (Sub)Types of surgery. Reported as n (%), unless otherwise stated.

Type of Surgery	N of Patients (%)
**Isolated Uvulopalatopharyngoplasty (UPPP)**	321 (6.9)
+ tonsillectomy	46 (1.0)
+ turbinate reduction	14 (0.3)
+ tongue radiofrequency ablation (RFA) + tonsillectomy	1 (0.02)
+ turbinate reduction + tongue RFA	3 (0.06)
+ tongue RFA	2 (0.04)
+ turbinate reduction + tonsillectomy	7 (0.2)
**Isolated Palatopharyngoplasty (PPP)**	1161 (25)
+ tonsillectomy	887 (19)
+ tonsillectomy + turbinate reduction	306 (6.6)
+ tonsillectomy + tongue RFA	70 (1.5)
+ tonsillectomy + turbinate reduction + tongue RFA	43 (0.9)
+ tonsillectomy + hyoid myotomy and suspension + turbinate reduction	5 (0.1)
+ turbinate reduction + sinus surgery	27 (0.6)
+ tongue RFA	76 (1.6)
+ turbinate reduction	409 (8.8)
+ sinus surgery	22 (0.5)
+ tonsillectomy + sinus surgery	9 (0.2)
+ turbinate reduction + tongue RFA	41 (0.9)
+ tonsillectomy + sinus surgery + turbinate reduction	9 (0.2)
+ tonsillectomy + turbinate reduction + sinus surgery + tongue RFA	2 (0.04)
+ turbinate reduction + sinus surgery + tongue RFA	1 (0.02)
+ sinus surgery + turbinate reduction + tonsillectomy	2 (0.04)
+ sinus surgery + tongue RFA	1 (0.02)
+ sinus surgery + tonsillectomy + tongue RFA	1 (0.02)
**Isolated Tonsillectomy**	578 (12)
+ turbinate reduction	85 (1.8)
+ turbinate reduction + sinus surgery	7 (0.2)
+ uvulectomy	76 (1.6)
+ uvulectomy + sinus surgery	2 (0.04)
+ turbinate reduction + uvulectomy	18 (0.4)
+ uvulectomy + tongue RFA	3 (0.06)
+ turbinate reduction + tongue RFA	1 (0.02)
+ tongue RFA	3 (0.06)
+ sinus surgery	2 (0.04)
**Isolated Uvulectomy**	31 (0.7)
+ turbinate reduction	14 (0.3)
+ sinus surgery + turbinate reduction	2 (0.04)
+ sinus surgery	1 (0.02)
**Isolated Partial Glossectomy**	13 (0.3)
+ PPP	35 (0.8)
+ PPP + turbinate reduction	6 (0.1)
+ turbinate reduction	4 (0.09)
+ PPP + tonsillectomy	28 (0.6)
+ tonsillectomy + PPP + turbinate reduction	7 (0.2)
+ tonsillectomy + turbinate reduction	3 (0.06)
+ tonsillectomy	2 (0.04)
+ hyoid myotomy and suspension	1 (0.02)
**Isolated Genioglossus Advancement**	0 (0.0)
+ maxillomandibular advancement	15 (0.3)
+ PPP + turbinate reduction	1 (0.02)
+ PPP + tonsillectomy	3 (0.06)
+ maxillomandibular advancement + sinus surgery + turbinate reduction	1 (0.02)
+ maxillomandibular advancement + PPP	4 (0.09)
+ maxillomandibular advancement + PPP + tonsillectomy	2 (0.04)
+ maxillomandibular advancement + PPP + turbinate reduction	2 (0.04)
+ maxillomandibular advancement + turbinate reduction	1 (0.02)
+ turbinate reduction	1 (0.02)
+ PPP	1 (0.02)
**Isolated Maxillomandibular Advancement**	25 (0.5)
**Isolated Hyoid Myotomy and Suspension**	35 (0.8)
+ tonsillectomy	6 (0.1)
+ PPP	26 (0.6)
+ tongue RFA	3 (0.06)
+ PPP + turbinate reduction	10 (0.2)
+ PPP + partial glossectomy	1 (0.02)
+ tongue RFA + turbinate reduction	4 (0.09)
+ PPP + tonsillectomy	7 (0.2)
+ PPP + tongue RFA	2 (0.04)
+ turbinate reduction	1 (0.02)
**Procedures Including Craniofacial Osteotomies**	32 (0.7)
**Procedures Including Epiglottidectomy**	16 (0.3)
**Procedures Including Tracheostomy**	18 (0.4)
**Other**	57 (1.2)

**Table 4:** Operative and postoperative outcomes for all patients undergoing head and neck OSA surgery. Reported as n (%), unless otherwise stated.

Outcome	Patients (n = 4662)
Length of Hospital Stay, mean days ± SD	0.9 ± 2.0
Operative Time, mean minutes ± SD	66 ± 54
**Any Complication**	292 (6.3)
**Mortality within 30 days**	2 (0.04)
**Reoperation**	163 (3.5)
**Readmission**	100 (2.1)
**Unplanned Readmission**	99 (2.1)
**Surgical Complication**	48 (1.0)
Superficial Incisional Infection	26 (0.6)
Deep Incisional Infection	4 (0.09)
Organ-Space Infection	8 (0.2)
Dehiscence	10 (0.2)
**Medical Complication**	55 (1.2)
Pneumonia	21 (0.5)
Reintubation	17 (0.4)
Pulmonary Embolism	4 (0.09)
Ventilator > 48 h	10 (0.2)
Myocardial Infarction	1 (0.02)
Cardiac Arrest Requiring CPR	1 (0.02)
DVT or Thrombophlebitis	5 (0.1)
Urinary Tract Infection	11 (0.2)
Septic Shock	1 (0.02)
Sepsis	8 (0.2)
**Discharge destination**	
Home	3956 (85)
Not Home	16 (0.3)
Other or Unknown	8 (0.2)

**Table 5:** Distribution of procedures with the type-specific occurrence of any complication.

Type of Surgery	Total	Any Complication	Any Complication (Total %)
**Uvulopalatopharyngoplasty (UPPP), of which:**			
Isolated	321	13	4.0
+ tonsillectomy	46	3	6.5
+ turbinate reduction	14	1	7.1
+ tongue radiofrequency ablation (RFA)	2	0	0.0
+ turbinate reduction + tonsillectomy	7	0	0.0
+ turbinate reduction + tongue RFA	3	0	0.0
+ tongue RFA + tonsillectomy	1	0	0.0
**Palatopharyngoplasty (PPP), of which:**			
Isolated	1161	65	5.6
+ tonsillectomy	887	57	6.4
+ tonsillectomy + turbinate reduction	306	18	5.9
+ tonsillectomy + tongue RFA	70	9	13
+ tonsillectomy + turbinate reduction + tongue RFA	43	0	0.0
+ tonsillectomy + hyoid myotomy and suspension + turbinate reduction	5	1	20
+ turbinate reduction + sinus surgery	27	2	7.4
+ tongue RFA	76	5	6.6
+ turbinate reduction	409	18	4.4
+ sinus surgery	22	0	0.0
+ tonsillectomy + sinus surgery	9	2	22
+ turbinate reduction + tongue RFA	41	4	9.8
+ tonsillectomy + sinus surgery + turbinate reduction	9	1	11
+ tonsillectomy + turbinate reduction + sinus surgery + tongue RFA	2	0	0.0
+ turbinate reduction + sinus surgery + tongue RFA	1	0	0.0
+ sinus surgery + turbinate reduction + tonsillectomy	2	0	0.0
+ sinus surgery + tongue RFA	1	0	0.0
+ sinus surgery + tonsillectomy + tongue RFA	1	0	0.0
**Tonsillectomy, of which:**			
Isolated	578	50	8.7
+ turbinate reduction	85	4	4.7
+ turbinate reduction + sinus surgery	7	0	0.0
+ uvulectomy	76	4	5.4
+ uvulectomy + sinus surgery	2	0	0.0
+ turbinate reduction + uvulectomy	18	2	11
+ uvulectomy + tongue RFA	3	0	0.0
+ turbinate reduction + tongue RFA	1	0	0.0
+ tongue RFA	3	0	0.0
+ sinus surgery	2	0	0.0
**Uvulectomy, of which:**			
Isolated	31	0	0.0
+ turbinate reduction	14	2	14
+ sinus surgery + turbinate reduction	2	0	0.0
+ sinus surgery	1	0	0.0
**Partial Glossectomy, of which:**			
Isolated	13	0	0.0
+ PPP	35	2	5.7
+ PPP + turbinate reduction	6	1	17
+ turbinate reduction	4	0	0.0
+ PPP + tonsillectomy	28	3	11
+ tonsillectomy + PPP + turbinate reduction	7	0	0.0
+ tonsillectomy + turbinate reduction	3	0	0.0
+ tonsillectomy	2	0	0.0
+ hyoid myotomy and suspension	1	0	0.0
**Genioglossus Advancement, of which:**			
Isolated	0	0	0.0
+ maxillomandibular advancement	15	2	13
+ PPP + turbinate reduction	1	0	0.0
+ PPP + tonsillectomy	3	0	0.0
+ maxillomandibular advancement + sinus surgery + turbinate reduction	1	0	0.0
+ maxillomandibular advancement + PPP	4	0	0.0
+ maxillomandibular advancement + PPP + tonsillectomy	2	1	50
+ maxillomandibular advancement + PPP + turbinate reduction	2	1	50
+ maxillomandibular advancement + turbinate reduction	1	0	0.0
+ turbinate reduction	1	0	0.0
+ PPP	1	0	0.0
**Isolated Maxillomandibular Advancement**	25	2	8.0
**Hyoid Myotomy and Suspension, of which:**			
Isolated	35	3	8.6
+ tonsillectomy	6	0	0.0
+ PPP	26	3	12
+ tongue RFA	3	0	0.0
+ PPP + turbinate reduction	10	1	10
+ PPP + partial glossectomy	1	0	0.0
+ tongue RFA + turbinate reduction	4	1	25
+ PPP + tonsillectomy	7	0	0.0
+ PPP + tongue RFA	2	0	0.0
+ turbinate reduction	1	0	0.0
**Procedures Including Craniofacial Osteotomies**	32	2	6.3
**Procedures Including Epiglottidectomy**	16	0	0.0
**Procedures Including Tracheostomy**	18	4	22
**Other**	57	5	8.8

**Table 6:** Risk-associated factors for complications. Reported as n (%), unless otherwise stated. Statistically significant *p*-values are highlighted in bold.

	Any Complication			Surgical Complication			Medical Complication		
Characteristic	Yes (n = 292)	No (n = 4370)	*p*-Value	Yes (n = 48)	No (n = 4614)	*p*-Value	Yes (n = 55)	No (n = 4607)	*p*-Value
**Demographics**									
**Sex**			**0.03**			0.20			0.36
Female (n)	64 (22)	1209 (28)		9 (19)	1264 (27)		18 (33)	1255 (27)	
Male (n)	228 (78)	3160 (72)		39 (81)	3349 (73)		37 (67)	3351 (73)	
Age, mean ± SD	41 ± 13	42 ± 13	0.44	46 ± 13	42 ± 13	**0.04**	45 ± 13	42 ± 13	0.05
BMI, mean ± SD	34 ± 8	33 ± 7	**0.01**	32 ± 6	33 ± 7	0.46	38 ± 9	33 ± 7	**<0.0001**
**Race**			0.30			**0.02**			0.66
American Indian or Alaskan Native	2 (0.7)	26 (0.6)		0 (0)	28 (0.1)		0 (0)	28 (0.6)	
Asian	23 (7.9)	221 (5.1)		2 (4.2)	242 (5.2)		3 (5.5)	241 (5.2)	
Native Hawaiian or Pacific Islander	4 (1.4)	47 (1.1)		1 (2.1)	50 (1.1)		0 (0)	51 (1.1)	
Black or African American	32 (11)	512 (12)		3 (6.3)	541 (12)		10 (18)	534 (12)	
White	173 (59)	2806 (64)		23 (48)	2956 (64)		32 (58)	2947 (64)	
Other or unknown	55 (19)	749 (17)		17 (35)	787 (17)		10 (18)	794 (17)	
**Setting**			**<0.0001**			**0.0002**			**<0.0001**
Outpatient	167 (57)	3113 (71)		22 (46)	3258 (71)		24 (44)	3256 (71)	
Inpatient	125 (43)	1257 (29)		26 (54)	1356 (29)		31 (56)	1351 (29)	
**Preop health and comorbidities**									
Diabetes	45 (15)	424 (9.7)	**0.002**	6 (13)	463 (10)	0.48	15 (27)	454 (9.9)	**<0.0001**
Insulin-treated diabetes	15 (5.1)	122 (2.8)	**0.02**	2 (4.2)	135 (2.9)	0.65	5 (9.1)	132 (2.9)	**0.02**
COPD	8 (2.7)	52 (1.2)	0.05	4 (8.3)	56 (1.2)	**0.003**	2 (3.6)	58 (1.3)	0.16
CHF	1 (0.3)	4 (0.09)	0.28	0 (0)	5 (0.1)	>0.99	1 (1.8)	4 (0.09)	0.06
Obesity	195 (67)	2714 (62)	0.11	29 (60)	2880 (62)	0.78	45 (82)	2864 (62)	**0.003**
Hypertension	106 (36)	1329 (30)	**0.03**	18 (38)	1417 (31)	0.31	21 (38)	1414 (31)	0.23
Dyspnea	21 (7.2)	208 (4.8)	0.06	6 (13)	223 (4.8)	**0.03**	6 (11)	223 (4.8)	0.05
Current smoker	52 (18)	690 (16)	0.36	7 (15)	735 (16)	>0.99	10 (18)	732 (16)	0.64
Corticosteroid use	7 (2.4)	73 (1.7)	0.35	2 (4.2)	78 (1.7)	0.20	4 (7.3)	76 (1.6)	**0.01**
Wound infection	2 (0.7)	11 (0.3)	1.19	0 (0)	13 (0.3)	>0.99	1 (1.8)	12 (0.3)	0.14
**ASA physical status classification score**			**<0.0001**			0.48			**0.0006**
1—No disturbance	13 (4.5)	204 (4.7)		4 (8.3)	213 (4.6)		1 (1.8)	216 (4.7)	
2—Mild disturbance	149 (51)	2501 (57)		24 (50)	2626 (57)		19 (35)	2631 (57)	
3—Severe disturbance	119 (41)	1625 (37)		19 (40)	1725 (37)		33 (60)	1711 (37)	
4—Life-threatening	10 (3.4)	35 (0.8)		1 (2.1)	44 (1.0)		2 (3.6)	43 (0.9)	
**Wound class**			0.81			0.82			0.22
1—Clean	14 (4.8)	165 (3.8)		2 (4.2)	177 (3.8)		4 (7.3)	175 (3.8)	
2—Clean/Contaminated	272 (93)	4124 (94)		46 (96)	4350 (94)		50 (91)	4346 (94)	
3—Contaminated	4 (1.4)	59 (1.4)		0 (0)	63 (1.4)		0 (0)	63 (1.4)	
4—Dirty/Infected	2 (0.7)	22 (0.5)		0 (0)	24 (0.5)		1 (1.8)	23 (0.5)	
**Functional Status**			0.08			0.44			**0.03**
Independent	285 (98)	4321 (99)		47 (98)	4559 (99)		52 (95)	4554 (99)	
Partially or totally dependent	7 (2.4)	49 (1.1)		1 (2.1)	55 (1.2)		3 (5.5)	53 (1.2)	

ASA, American Society of Anesthesiology.

**Table 7:** Multivariable assessment of any, surgical, and medical complication occurrences for all patients undergoing head and neck OSA surgery.

Risk-Associated Factors	OR	95% CI	*p*-Value
**Any complications**			
Sex (female)	−0.02	−0.04–−0.01	0.003
Diabetes	0.03	0.00–0.05	0.03
ASA physical status classification score (≥4)	0.12	0.05–0.20	0.001
**Surgical complications**			
Race (White)	−0.02	−0.02–−0.01	<0.0001
Race (Black or African American)	−0.02	−0.03–−0.01	0.004
COPD	0.05	0.02–0.07	0.0006
**Medical complications**			
Diabetes	0.02	0.01–0.03	0.003
History of CHF	0.15	0.05–0.24	0.004
Corticosteroid use	0.03	0.01–0.06	0.006
Underweight; BMI < 18.5	0.08	0.01–0.14	0.02
Extreme Obesity Class 3; BMI > 40	0.01	0.00–0.02	0.02

ASA, American Society of Anesthesiology.

**Table 8:** Multivariable assessment of any, surgical, and medical complication occurrences with regard to different types of surgeries.

Risk-Associated Factors	OR	95% CI	*p*-Value
**Any complications**			
Isolated tonsillectomy	0.05	0.02–0.09	0.003
Procedures including tracheostomy	0.12	0.00–0.25	0.04
PPP + tonsillectomy + tongue RFA	0.09	0.02–0.15	0.007
PPP + tonsillectomy + sinus surgery	0.17	0.01–0.33	0.03
Genioglossus advancement + maxillomandibular advancement + PPP + tonsillectomy	0.45	0.11–0.78	0.009
Genioglossus advancement + maxillomandibular advancement + turbinate reduction	0.96	0.48–1.43	<0.0001
**Surgical complications**			
Genioglossus advancement + maxillomandibular advancement	0.06	0.01–0.11	0.03
Genioglossus advancement + maxillomandibular advancement + turbinate reduction	0.99	0.79–1.18	<0.0001
Hyoid myotomy and suspension	0.07	0.04–0.11	<0.0001
Hyoid myotomy and suspension + PPP	0.06	0.02–0.10	0.001
Hyoid myotomy and suspension + tongue RFA + turbinate reduction	0.24	0.15–0.34	<0.0001
**Medical complications**			
Procedures including tracheostomy	0.06	0.00–0.11	0.04
Genioglossus advancement + maxillomandibular advancement	0.06	0.00–0.11	0.04
PPP + tonsillectomy + tongue RFA	0.03	0.00–0.06	0.04
PPP + tonsillectomy + sinus surgery	0.09	0.02–0.17	0.01
PPP + tonsillectomy + sinus surgery + turbinate reduction	0.11	0.03–0.18	0.004
Hyoid myotomy and suspension + PPP + turbinate reduction	0.09	0.02–0.16	0.01
UPPP + turbinate reduction	0.07	0.01–0.13	0.02

PPP: palatopharyngoplasty; RFA: radiofrequency ablation; UPPP, uvulopalatopharyngoplasty.

**Table 9:** Preoperative lab values for complications. Statistically significant *p*-values are highlighted in bold.

	Any Complication			Surgical Complication			Medical Complication			
Characteristic	Yes (n = 292)	No (n = 4370)	*p*-Value	Yes (n = 48)	No (n = 4614)	*p*-Value	Yes (n = 55)	No (n = 4607)	*p*-Value	Reference Range
Serum sodium (mmol/L)	139.5 (2.7)	139.4 (2.5)	0.45	139.0 (3.2)	139.4 (2.5)	0.51	139.6 (2.3)	139.4 (2.5)	0.57	135–145 mmol/L
BUN (mg/dL)	15.3 (8.3)	15.1 (5.4)	0.64	16.2 (4.8)	15.1 (5.6)	0.43	16.5 (12.0)	15.1 (5.4)	0.14	8–25 mg/dL
Creatinine (g/D)	0.9 (0.5)	1.0 (0.5)	0.65	1.0 (0.3)	1.0 (0.5)	0.79	1.0 (0.5)	1.0 (0.5)	0.61	F 0.6–1.8, M 0.8–2.4 g/D
Serum albumin (g/dL)	4.2 (0.5)	4.3 (0.4)	0.38	4.3 (0.5)	4.2 (0.4)	0.78	4.1 (0.4)	4.3 (0.4)	0.16	3.1–4.3 g/dL
Total bilirubin (mg/dL)	0.7 (0.9)	0.6 (0.5)	0.07	0.6 (0.3)	0.6 (0.6)	0.91	0.7 (0.5)	0.6 (0.6)	0.40	0–1 mg/dL
SGOT (U/L)	28.4 (15.4)	27.1 (25.5)	0.68	20.0 (4.7)	27.3 (25.0)	0.35	25.3 (10.2)	27.3 (25.1)	0.74	F 9–25, M 10–40 U/L
Alkaline phosphatase (U/L)	81.5 (32.8)	74.4 (23.5)	**0.02**	70.3 (18.9)	75.0 (24.3)	0.57	94.1 (43.5)	74.6 (23.7)	**0.001**	F 30–100 U/L
WBC × 10^3^/mm^3^	7.8 (2.6)	7.4 (2.8)	0.09	7.2 (2.1)	7.5 (2.8)	0.66	8.1 (2.2)	7.4 (2.8)	0.15	4.5–11 × 10^3^/mm^3^
Hematocrit (% of RBCs)	43.0 (4.9)	42.8 (4.2)	0.64	43.8 (3.9)	42.8 (4.2)	0.26	42.4 (4.5)	42.8 (4.2)	0.58	F 36.0–46.0%, M 37.0–49.0% of RBCs
Platelet count × 10^3^/µL	248.2 (62.9)	250.4 (64.3)	0.68	240.7 (46.1)	250.4 (64.4)	0.48	242.3 (55.3)	250.4 (64.4)	0.47	130–400 × 10^3^/µL
PTT (s)	30.2 (3.7)	29.3 (5.2)	0.20	31.7 (4.7)	29.4 (5.1)	0.19	29.8 (4.7)	29.4 (5.1)	0.77	25–35 s
INR of PT values	1.0 (0.1)	1.0 (0.3)	0.86	1.0 (0.1)	1.0 (0.3)	0.89	1.0 (0.1)	1.0 (0.3)	0.95	<1.1

BUN, blood urea nitrogen; SGOT, serum glutamic-oxaloacetic transaminase; WBC, white blood cell; PTT, partial thromboplastin time; INR, international normalized ratio; PT, prothrombin time; s, seconds; SD, standard deviation; RBC, red blood cell.

## Data Availability

Restrictions apply to the availability of these data. Data were obtained from the American College of Surgeons—National Surgical Quality Improvement Program. The application can be submitted at https://accreditation.facs.org/programs/nsqip.

## Supplementary Materials

The following supporting information can be downloaded at: https://www.mdpi.com/article/10.3390/jcm11247371/s1. Table S1: Demographics and comorbidities of all patients who underwent isolated uvulopalatopharyngoplasty (UPPP), palatopharyngoplasty (PPP), and tonsillectomy. Table S2: Comparison of complication rates in the different procedures when the procedures were performed as isolated procedures and when they were performed in combination with other procedures. Table S3: Comparison of complication rates in the different procedures when the procedures were performed as isolated procedures. Figure S1: Frequency of complications stratified by BMI classes.
