# Supplementary material for: 30-Day Postoperative Outcomes in Adults with Obstructive Sleep Apnea Undergoing Upper Airway Surgery
Source: J Clin Med. 2022 Dec 12;11(24):7371. doi: 10.3390/jcm11247371 (PMC9783895; doi:10.3390/jcm11247371)
Supplement: Supplementary file 1 [file jcm-11-07371-s001.zip › jcm-2015176-supplementary.pdf]

SUPPLEMENTARY MATERIAL

Supplementary Figure S1. Frequency of complications stratified by BMI classes. Strikingly, the vast majority of complications occurred in patients with pathological BMI values. It is further noteworthy, that among the underweight cohort (n=11), three cases (27%) of medical complications occurred.

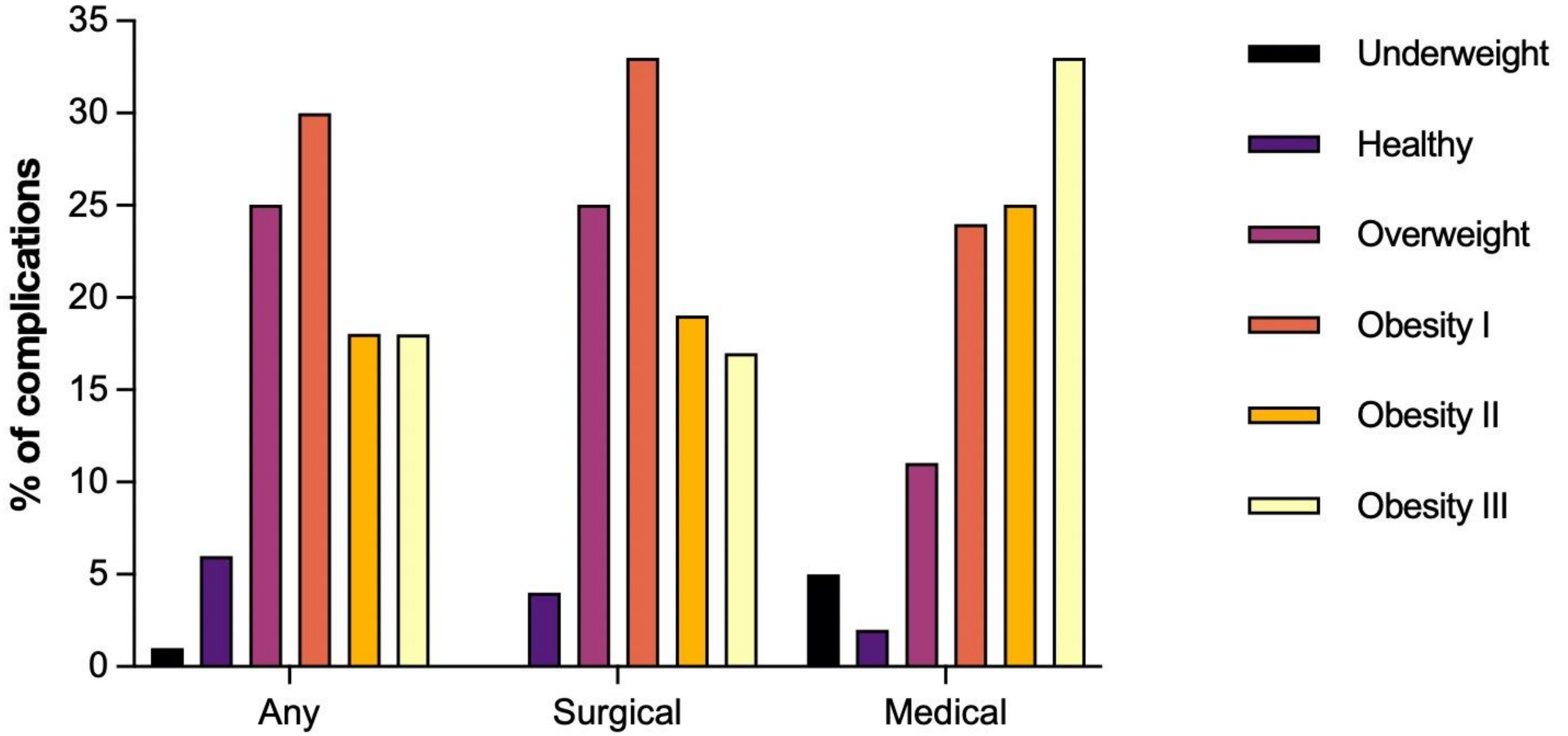

Underweight: BMI <18.5; Normal weight: BMI 18.5-24.9; Overweight: BMI 25-29.9; Obesity I: BMI 30-34.9; Obesity II: BMI 35-39.9; Obesity III BMI > 40 kg/m^2

Supplementary Table S1. Demographics and comorbidities of all patients who underwent isolated uvulopalatopharyngoplasty (UPPP), palatopharyngoplasty (PPP), and tonsillectomy. Generally, the UPP and PPP cohorts showed highly similar patient characteristics. In comparison, patients who underwent tonsillectomy were on average about 10 years younger, more frequently female, and tend to be healthier (as evidenced by a lower prevalence of comorbidities, such as hypertension, diabetes, or smoking status).

Characteristic	UPPP (n=321)	PPP (n=1,161)	Tonsillectomy (n=578)
**Demographics**			
Sex			
Female (n)	74 (23)	309 (27)	256 (44)
Male (n)	247 (77)	852 (73)	322 (56)
Age, mean ± SD	44 ± 11	44 ± 12	32 ± 12
BMI, mean ± SD	33 ± 7.2	33 ± 7.3	33 ± 8.1
**Race**			
American Indian or Alaskan native	0 (0.0)	3 (0.2)	4 (0.7)
Asian	7 (2.2)	68 (5.9)	30 (5.1)
Native Hawaiian or Pacific Islander	0 (0.0)	10 (0.9)	5 (0.9)
Black or African American	31 (9.7)	133 (11)	69 (12)
White	213 (66)	741 (64)	352 (61)
Other or unknown	70 (22)	204 (18)	118 (20)
**Preoperative health and comorbidities**			
Diabetes	32 (10)	109 (9.3)	40 (6.9)
Insulin treated diabetes	5 (1.6)	26 (2.2)	17 (2.9)
COPD	7 (2.2)	14 (1.2)	6 (1.0)
CHF	0 (0.0)	2 (0.2)	0 (0.0)
Obesity	194 (60)	724 (62)	362 (63)
Hypertension	107 (33)	390 (33)	113 (20)
Dyspnea	25 (7.8)	50 (4.3)	21 (3.6)
Current smoker	63 (20)	200 (17)	98 (17)
Corticosteroid use	1 (0.3)	16 (1.4)	10 (1.7)
Wound infection	1 (0.3)	3 (0.2)	0 (0.0)
**ASA physical status classification score**			
1 – No disturbance	18 (5.6)	61 (5.2)	57 (6.4)
2 – Mild disturbance	211 (66)	745 (64)	367 (63)
3 – Severe disturbance	88 (27)	343 (29)	169 (29)
4 – Life-threatening	3 (0.9)	11 (0.9)	4 (0.7)
**Wound class**			
1 – Clean	0 (0.0)	27 (2.3)	14 (2.4)
2 – Clean/Contaminated	317 (99)	1115 (96)	555 (96)
3 – Contaminated	2 (0.6)	10 (0.8)	6 (1.0)
4 – Dirty/Infected	2 (0.6)	8 (0.6)	3 (0.5)
**Functional Status**			
Independent	320 (100)	1151 (99)	570 (99)
Partially or Totally Dependent	1 (0.3)	10 (0.9)	8 (1.4)

ASA, American Society of Anesthesiology

Supplementary Table S2. Comparison of complication rates in the different procedures when the procedures were performed as isolated procedures and when they were performed in combination with other procedures. No significant differences were noted in the complication rates between isolated and combined procedures. Numbers are presented as n and (%).

	Isolated		Combined		
Procedure	No complications	Complications	No complications	Complications	P value
UPPP	308	13	69	4	0.53
PPP	1096	65	1794	117	0.58
Tonsillectomy	528	50	187	10	0.12
Uvulectomy	31	0	15	2	0.14
Partial glossectomy	13	0	80	6	>0.99
Hyoid Myotomy and Suspension	32	3	55	5	>0.99

UPPP, uvulopalatopharyngoplasty; PPP, palatopharyngoplasty.

Supplementary Table S3. Comparison of complication rates in the different procedures when the procedures were performed as isolated procedures. Isolated tonsillectomy was associated with a significantly higher rate of complications than isolated UPPP and isolated PPP. Procedures involving tracheostomy had the highest rate of complications, significantly higher than isolated UPPP, isolated PPP, and isolated uvulectomy. The numbers presented are p values.

Isolated procedure	PPP	Tonsillectomy	Uvulectomy	Partial glossectomy	Maxillomandibular Advancement	Hyoid Myotomy and Suspension	Craniofacial osteotomies	Epiglottidectomy	Tracheostomy
UPPP	0.32	**0.009**	0.62	>0.99	0.30	0.20	0.64	>0.99	**0.009**
PPP		**0.02**	0.41	>0.99	0.65	0.45	0.70	>0.99	**0.02**
Tonsillectomy			0.10	0.62	>0.99	>0.99	>0.99	0.38	0.07
Uvulectomy				>0.99	0.24	0.19	0.49	>0.99	**0.01**
Partial glossectomy					0.54	0.55	>0.99	>0.99	0.12
MMA						>0.99	>0.99	0.51	0.22
HM&S							>0.99	0.54	0.21
CO								0.55	0.17
Epiglottidectomy									0.11

UPPP, uvulopalatopharyngoplasty; PPP, palatopharyngoplasty; MMA, Maxillomandibular Advancement; HM&S, Hyoid Myotomy and Suspension; CO, Craniofacial osteotomies.
